# Supplementary material for: Increasing Influenza Vaccination Rates among Patients with Type 2 Diabetes Mellitus in Chongqing, China: A Cross-Sectional Analysis Using Behavioral and Social Drivers Tools
Source: Vaccines (Basel). 2024 Aug 8;12(8):898. doi: 10.3390/vaccines12080898 (PMC11360589; doi:10.3390/vaccines12080898)
Supplement: Supplementary file 1 [file vaccines-12-00898-s001.zip › vaccines-3108266-supplementary.pdf]

Influenza Vaccination Willingness and Influencing Factors Survey for Patients with Type 2 Diabetes Mellitus in Chongqing

Dear resident, to better understand the influenza vaccination status, willingness, and influencing factors among patients with T2DM and provide a basis for formulating scientific vaccination policies, the Chongqing Center for Disease Control and Prevention, in collaboration with Chongqing Medical University, is conducting this survey. Your cooperation is crucial for improving influenza prevention and control among key populations like diabetes patients. Thank you for your participation!

Informed Consent: I have read the above information, fully considered it, and voluntarily participate in this survey. ☐ Yes ☐ No (End of Survey)

Part I: Basic Information

1.Your sex: ☐ Male ☐ Female

2.Your date of birth: _______________

3.Your ethnicity: ☐ Han ☐ Minority ethnicity

4.Your education level: ☐ Primary school and below ☐ Junior high school ☐ Senior high school or equivalent ☐ College/bachelor's degree or above

5.Your residence: ☐ Urban ☐ Rural

6.Your occupation: ☐ Office worker ☐ Businessman

☐ Worker/Farmer ☐ Retiree ☐ The unemployed

7.Your marital status: ☐ Single ☐ Married ☐ Widowed ☐ Divorced

8.Your average monthly household income per capita:

☐ <2000 RMB ☐ 2000-5000 RMB ☐ >5000 RMB

9.Your height is ____ (unit: cm) **weight** is ____ (unit: kg) (Please keep one decimal place, e.g., height 173.5 cm, weight 70.5 kg)

10.You were diagnosed with diabetes in ___ year (Please write: year, e.g., 2018)

11.Do you have any complications of diabetes mellitus? ☐ Yes ☐ No

12.Do you have any other chronic diseases? ☐ Yes ☐ No

Part II: Status of Influenza Illness and Vaccination

13.During the 2022/2023 influenza season, have you diagnosed with influenza?

☐ Yes, severe ☐ Yes, mild ☐ None

14.During the 2022/2023 influenza season, did you receive an influenza vaccination?

☐ Yes ☐ No (Skip to question 18)

15.Where did you get vaccinated against influenza?

☐ Hospital ☐ Local centers for disease control and prevention ☐ Community health centers

16.Did you experience any adverse reactions after your most recent influenza vaccination?

☐ Yes ☐ No

17.Your payment of influenza vaccination?

☐ Self-paid ☐ Employer paid ☐ Medical insurance

Part III: Knowledge and practice of influenza vaccine among T2DM patients

18.Learning about influenza prevention and control ☐ Yes ☐ No

19.Acquiring knowledge about influenza vaccination ☐ Yes ☐ No

20.Recognizing influenza vaccination as the most effective method for preventing influenza

☐ Yes ☐ No

21.The priority groups for influenza vaccination

	Yes	No
Healthcare workers		
Children 6-59 months of age		
Adults ≥60 years of age		
Pregnant women		
People living in nursing homes or welfare homes and staff who take care of vulnerable, at-risk individual		
People who work in nursery institutions, primary and secondary schools, and supervision places		

Participants and support personnel for large-scale events		
Individuals with chronic respiratory diseases		
Individuals with high blood pressure		
Individuals with diabetes mellitus		

Part IV: Factors Influencing Vaccination Willingness

22.Would you be willing to receive influenza vaccination during this winter-spring season (2023/2024 influenza season)?

☐ Definitely yes ☐ Definitely not (Please skip to question 24) ☐ Not sure (Please skip to question 24)

23. Driving Factors

	**Disagree**	**Neutral**	**Agree**
**Thinking and feeling**			
Worry about spreading influenza to family members /friends			
Worry about influenza worsening diabetes			
Worry about influenza affecting work and life			
Safety and reliability of IV			
Effectiveness of IV			
**Social processes**			
Recommendations by friends/ family members			
Recommendations by community/ workplace			
Recommendations by doctors			
**Practical issues**			
Affordable vaccine price			
Convenient vaccination			
Sufficient IV			

24. Hesitancy Factors

	Disagree	Neutral	Agree
**Thinking and feeling**			
Influenza will not cause severe illness			
No benefits of influenza vaccination			
Worry about adverse reactions to IV			
Lack of safety in IV			
Ineffectiveness of IV			
Worry about contraindications			
**Social processes**			
No recommendations by friends/ family members			
No recommendations by community/ workplace			
No recommendations by doctors			
**Practical issues**			
High cost of IV			
Uncertainty about time of influenza vaccination			
Uncertainty about site of influenza vaccination			
Inconvenience of influenza vaccination site			
Influenza vaccination service is inconvenient			

25.If your local area offers free influenza vaccination for diabetes patients, would you be willing to get vaccinated?

☐ Yes ☐ No

26.Attention Check: Please choose "Somewhat Disagree" for this question to verify your attention.

☐ Strongly Disagree ☐ Somewhat Disagree ☐ Neutral ☐ Somewhat Agree ☐ Strongly Agree

Thank you very much for your participation!
